# Prevalence of Allergic Diseases across All Ages in Japan: A Nationwide Cross-Sectional Study Employing Designated Allergic Disease Medical Hospital Network

**DOI:** 10.31662/jmaj.2022-0218

**Published:** 2023-03-24

**Authors:** Yasunori Ito, Taisuke Kato, Koichi Yoshida, Kyohei Takahashi, Yuma Fukutomi, Mizuho Nagao, Tatsuki Fukuie, Hiroshi Matsuzaki, Minoru Gotoh, Akio Tanaka, Satoshi Konno, Junichiro Tezuka, Yosikazu Nakamura, Yuichi Adachi

**Affiliations:** 1 Pediatric Allergy Center, Nagano Children’s Hospital, Nagano, Japan; 2 Department of Pediatrics, University of Toyama, Toyama, Japan; 3 Department of Allergy, Tokyo Metropolitan Children’s Medical Center, Tokyo, Japan; 4 Department of Pediatrics, National Hospital Organization Sagamihara National Hospital, Kanagawa, Japan; 5 Clinical Research Center for Allergy and Rheumatology, National Hospital Organization Sagamihara National Hospital, Kanagawa, Japan; 6 Institute for Clinical Research, National Hospital Organization Mie National Hospital, Mie, Japan; 7 Allergy Center, National Center for Child Health and Development, Tokyo, Japan; 8 Department of Pediatrics, National Hospital Organization Fukuoka National Hospital, Fukuoka, Japan; 9 Department of Otorhinolaryngology, Nippon Medical School, Tokyo, Japan; 10 Department of Dermatology, Graduate School of Biomedical and Health Sciences Hiroshima University, Hiroshima, Japan; 11 Department of Respiratory Medicine, Faculty of Medicine, Hokkaido University, Hokkaido, Japan; 12 Department of Allergy and Pulmonology, Fukuoka Children’s Hospital, Fukuoka, Japan; 13 Division of Public Health, Center for Community Medicine, Jichi Medical University, Tochigi, Japan

**Keywords:** allergy, prevalence, allergic disease medical hospital, national cross-sectional study

## Abstract

**Introduction:**

Allergic diseases affect both children and adults, but generation-specific prevalence rates are unclear.

**Methods:**

An online questionnaire was used from December 2021 to January 2022 to survey the prevalence of allergic diseases among staff and their families of designated allergic disease medical hospitals in Japan. In this study, bronchial asthma (BA), atopic dermatitis (AD), food allergies (FAs), allergic rhinitis (AR), allergic conjunctivitis (AC), metal allergies (MAs), and drug allergies (DAs) were the allergic diseases surveyed.

**Results:**

In total, 18,706 individuals were surveyed (median age, 36 years; quartile range, 18-50). Allergic disease was reported in 62.2% of respondents. Across all ages, prevalence rates were as follows: BA (14.7%), AD (15.6%), FAs (15.2%), AR (47.4%), AC (19.5%), MAs (1.9%), and DAs (4.6%). The prevalence of BA and AR was higher in male children, whereas that of FAs and AC was higher in adult females. The prevalence of MAs and DAs peaked during adulthood and predominated among females.

**Conclusions:**

Our results suggest that approximately two-thirds of the Japanese population may have an allergic disease, with AR being the most prevalent.

## Introduction

Allergic diseases such as bronchial asthma (BA), atopic dermatitis (AD), food allergies (FAs), allergic rhinitis (AR), and allergic conjunctivitis (AC), with symptoms that involve multiple organs including respiratory and digestive organs and the skin, have a high prevalence in both children and adults.

Due to genetic or environmental factors, allergic diseases can arise, and their prevalence varies depending on ethnicity and culture. The prevalence of allergic diseases has been reported to vary significantly between different countries ^(1)^. In recent decades, environmental changes and advancements in treatments have significantly affected the prevalence of allergic diseases. Several surveys of the prevalence of allergic diseases have been conducted in Japan; however, in most of the reports, participants were limited to only children or adults, and not all allergic diseases have been included. Moreover, few cross-national studies of allergic disease prevalence have surveyed multiple allergic diseases across all ages. Surveys on the prevalence of allergic diseases are important to assess the effects of environmental and lifestyle changes on allergic diseases over time and across generations. It is known that during childhood allergic diseases develop dynamically over time in a phenomenon called the allergic march ^(2)^.

In 2016, the Basic Act on Allergic Disease Measures was enacted in Japan, and the government promotes the use of its comprehensive allergic disease measures ^(3)^. According to this act, each prefecture has designated allergic disease medical hospitals to provide medical care for patients with severe allergic diseases, educate the community about allergic diseases, and participate in epidemiological surveys that are conducted by the Japanese government. Thus, we carried out the first nationwide cross-sectional survey to evaluate, using the designated allergic disease medical hospital network, the prevalence of allergic diseases across all ages in Japan.

## Materials and Methods

### Study design

In this cross-sectional study, an online questionnaire was employed, targeting staff (physicians, nurses, laboratory technicians, pharmacists, and clerical workers) and their families at allergic disease medical hospitals. From December 2021 to January 2022, this survey was announced via a leaflet or e-mail to hospital staff. Those who were willing to participate in this survey responded to an online questionnaire.

### Ethical considerations

This study was conducted according to the principles laid out in the Helsinki Declaration and the ethical guidelines for medical and health research studies that involve human subjects outlined by the Ministry of Health, Labour, and Welfare of Japan. The study was approved by an ethics committee from Toyama University (ID: R2021089). All participants provided informed consent.

### Questionnaire and definitions

To administer the online questionnaire, we used the WEBCAS^Ⓡ^ system (WOW WORLD Inc.). The questionnaire was written in Japanese, and the participating staff answered the questions regarding their own and their family members’ history of allergic diseases. Questions included information concerning age, sex, occupation, work facility name, history of allergic diseases, and the prevalence of current symptoms of allergic diseases. Allergic diseases investigated in this survey were BA, AD, FAs, AR, AC, metal allergies (MAs), and DAs.

### Determining the prevalence of a history of allergic diseases

The respondents who answered “Yes” to the question “Have you (or your family members) ever been diagnosed by a physician for BA, AD, FAs, AR, and AC?” were defined as “diagnosed.” Those who answered “No, but I probably have the disease” were defined as “probably having the disease.” Respondents were considered to have AR if they had a history of perennial AR (pAR) or pollinosis (PO). We calculated the history rates of allergic diseases based on those diagnosed and those probably having the disease for all allergic diseases except for MAs and DAs. Concerning MAs and DAs, there were only two responses (Yes or No) to the question “Have you (or your family members) ever been diagnosed by a physician with MAs or DAs?” Those who answered “Yes” were defined as “diagnosed.”

### Determining the prevalence of current symptoms

The respondents who reported a history of BA, AD, AR, and AC were asked the following question: “Have you (or your family members) had any symptoms of the disease during the previous 12 months?” and “Have you (or your family members) been treated for the disease during the previous 12 months?” Those who answered “Yes” to at least one of these questions were defined as having “current symptoms.”

Regarding FAs, those who answered the question “Have you (or your family members) ever been diagnosed with FAs by a physician?” with either “Yes, there are foods that have been diagnosed as allergic and are still being removed” or “No, but there are foods that have been removed due to symptoms” were defined as having “current symptoms.”

### Response analysis and statistical analysis

There were 14 age group categories, namely, 12 categories every 5 years from 0 to 59 years, one category for respondents aged 60-69 years, and one category for respondents aged ≥70 years. Prevalence rates and the sex ratio for each disease were calculated within each of these age categories. We calculated 95% confidence intervals for a history of each disease. Prevalence rates between male and female respondents were compared using a chi-square test. A two-sided *P*-value of <5% was considered significant. For the statistical analysis, STATA-17 (StataCorp, Lakeway, TX, USA) software was employed.

## Results

### Overview of the respondents

Fifty-eight allergic disease medical hospitals cooperated in this survey (Table 1). Of the 7,078 respondents, 105 were excluded who either declined to answer certain questions or inputted errors or duplicate data; thus, 6,973 responses were included in the analysis (nurses, n = 2,620; physicians, n = 1,391; clerks, n = 1,295; medical technologists, n = 306; pharmacists, n = 263; and others, n = 1,098). The data were analyzed from 18,706 respondents, including the information they provided regarding their family members. The median age of the respondents was 36 years (quartile, 18-50; min-max: 0-102 years), comprising 8,175 males and 10,531 females. Among all respondents, 11,626 (62.2%) were determined to have some form of allergic disease according to our criteria (males, n = 4,974, 60.8%; females, n = 6,652, 63.2%).

**Table 1.** List of Survey Hospitals.

Hospital	Prefecture	Participants for analysis		Hospital	Prefecture	Participants for analysis
Hokkaido University Hospital	Hokkaido	229		Aichi Children’s Health and Medical Center	Aichi	75
Hirosaki University Hospital	Aomori	109		Fujita Health University Bantane Hospital	Aichi	926
NHO Morioka National Hospital	Iwate	109		Fujita Health University Hospital	Aichi	73
Tohoku University Hospital	Miyagi	23		NHO Mie National Hospital	Mie	383
Miyagi Children’s Hospital	Miyagi	32		Mie University Hospital	Mie	33
Yamagata University Hospital	Yamagata	101		Shiga Medical Center for Children	Shiga	122
Fukushima Medical University Hospital	Fukushima	58		Kansai Medical University Hospital	Osaka	90
Dokkyo Medical University Hospital	Tochigi	100		Osaka Habikino Medical Center	Osaka	424
Gunma University Hospital	Gunma	183		Osaka Red Cross Hospital	Osaka	201
Saitama Medical University Hospital	Saitama	116		Kobe University Hospital	Hyogo	301
Chiba University Hospital	Chiba	495		The Hospital of Hyogo College of Medicine	Hyogo	35
National Center for Child Health and Development	Tokyo	1,114		Hyogo Prefectural Kobe Children’s Hospital	Hyogo	39
Tokyo Medical and Dental University Medical Hospital	Tokyo	101		Nara Medical University Hospital	Nara	1,280
Tokyo Metropolitan Children’s Medical Center	Tokyo	309		Japanese Red Cross Wakayama Medical Center	Wakayama	290
NHO Sagamihara National Hospital	Kanagawa	295		Tottori University Hospital	Tottori	252
Kanagawa Children’s Medical Center	Kanagawa	23		Okayama University Hospital	Okayama	904
Niigata University Medical & Dental Hospital	Niigata	363		NHO Minami-Okayama Medical Center	Okayama	453
Toyama Prefectural Central Hospital	Toyama	88		Hiroshima University Hospital	Hiroshima	1,083
Toyama University Hospital	Toyama	840		Yamaguchi University Hospital	Yamaguchi	455
Kanazawa University Hospital	Ishikawa	741		Tokushima University Hospital	Tokushima	324
University of Fukui Hospital	Fukui	653		Kagawa University Hospital	Kagawa	20
University of Yamanashi Hospital	Yamanashi	434		Ehime University Hospital	Ehime	369
Shinshu University Hospital	Nagano	205		Kochi Medical School Hospital	Kochi	498
Nagano Children’s Hospital	Nagano	480		NHO Fukuoka National Hospital	Fukuoka	708
Gifu University Hospital	Gifu	486		Saga University Hospital	Saga	83
IUHW Atami Hospital	Shizuoka	3		Kumamoto University Hospital	Kumamoto	68
Juntendo University Shizuoka Hospital	Shizuoka	841		Oita University Hospital	Oita	427
Shizuoka General Hospital	Shizuoka	109		University of Miyazaki Hospital	Miyazaki	26
Hamamatsu Medical Center	Shizuoka	32		Kagoshima University Hospital	Kagoshima	92

### BA

**Figure 1.**
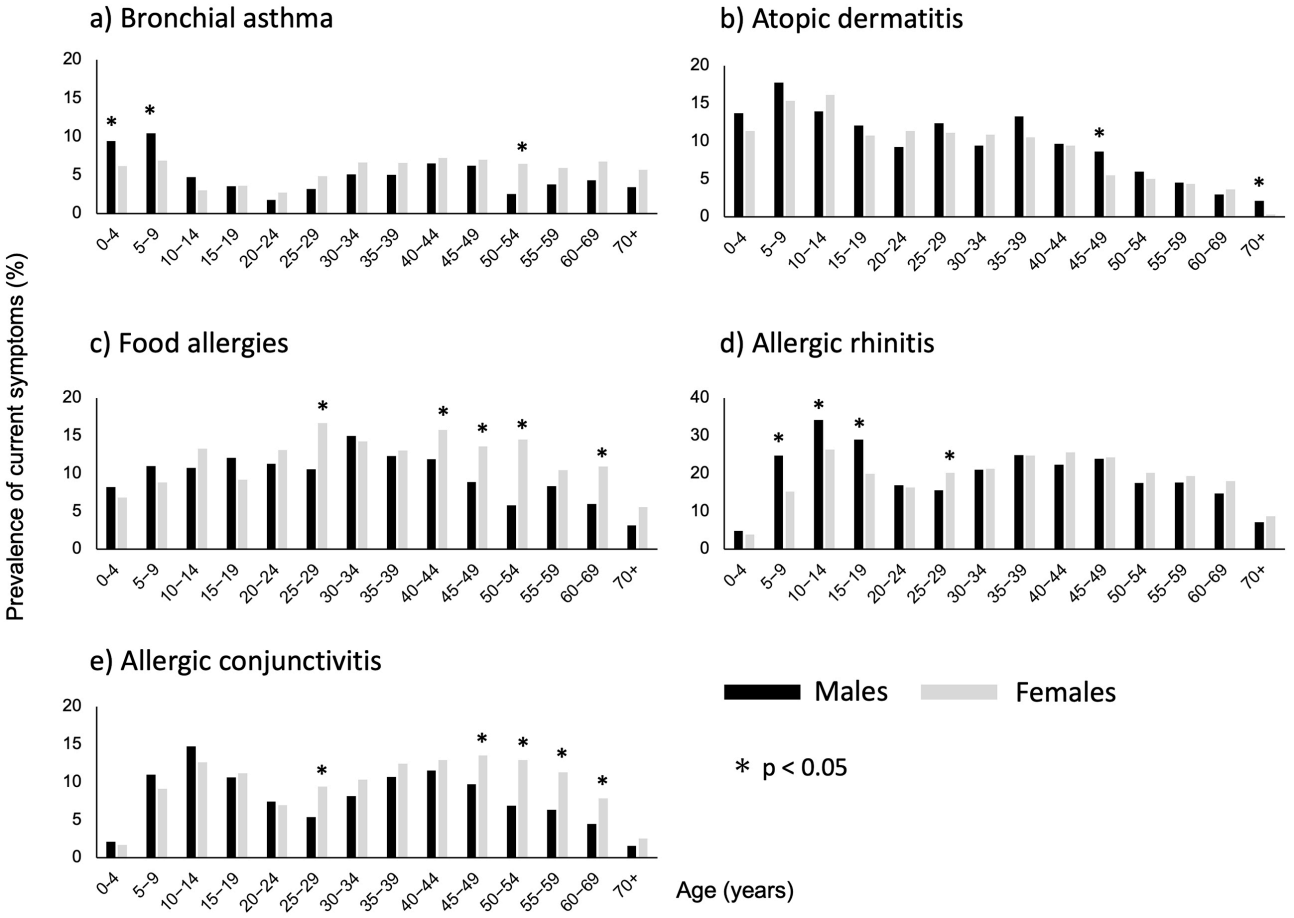
Prevalence of current symptoms of allergic diseases. The percentage of respondents who reported current symptoms of allergic diseases. a) Bronchial asthma, b) Atopic dermatitis, c) Food allergies, d) Allergic rhinitis, e) Allergic conjunctivitis *P*-values for sex differences in each age category were calculated using the chi-squared test.

The prevalence of a history of BA was 14.7% (those diagnosed, n = 2,420; those with a probable diagnosis, n = 327) (Table 2). The prevalence of current symptoms peaked in those aged 5-9 years (8.7%), decreased in those aged 20-29 years (2.4%), increased in adults aged ≥30 years, and remained approximately 5% higher in males aged 0-4 years and 5-9 years, and in females aged 50-54 years (*P* < 0.05; Figure 1-a).

**Table 2.** Prevalence of a History of Allergic Disease at Each Age.

Age	n	Male/ female	Bronchial asthma	Atopic dermatitis	Food allergy	Allergic rhinitis	Allergic conjunctivitis	Metal allergy	Drug allergy
Total	18,706	8175/10531	14.7% (14.2-15.2)	15.6% (15.0-16.1)	15.2% (14.7-15.7)	47.4% (46.6-48.1)	19.5% (18.9-20.1)	1.9% (1.7-2.1)	4.6% (4.3-4.9)
Age (years)									
0-4	1,254	656/598	9.5% (7.9-11.2)	13.5% (11.6-15.5)	13.0% (11.2-15.0)	7.6% (6.2-9.3)	2.7% (1.9-3.8)	0%	0.6% (0.3-1.3)
5-9	1,378	708/670	15.6% (13.7-17.6)	19.6% (17.5-21.8)	18.0% (16.0-20.1)	30.6% (28.1-33.1)	13.7% (11.9-15.6)	0%	1.4% (0.8-2.1)
10-14	1,256	631/625	14.3% (12.4-16.4)	19.7% (17.6-22.1)	19.3% (17.2-21.6)	49.8% (47.0-52.6)	21.6% (19.3-24.0)	0.2% (0-0.6)	1.4% (0.8-2.2)
15-19	980	479/501	18.4% (16.0-20.9)	16.9% (14.6-19.4)	16.9% (14.6-19.4)	53.4% (50.2-56.5)	21.0% (18.5-23.7)	0.2% (0-0.7)	3.0% (2.0-4.2)
20-24	1,068	336/732	13.8% (11.7-16.0)	18.4% (16.1-20.8)	18.4% (16.2-20.9)	55.6% (52.6-58.6)	19.4% (17.1-21.9)	1.0% (0.1-1.8)	3.6% (2.5-4.9)
25-29	1,544	501/1,043	16.7% (14.9-18.7)	21.0% (19.0-23.1)	19.4% (17.5-21.5)	58.0% (55.5-60.4)	20.5% (18.5-22.6)	1.7% (1.1-2.5)	4.2% (3.3-5.3)
30-34	1,510	648/862	17.0% (15.1-18.9)	19.5% (17.5-21.6)	17.7% (15.9-19.8)	54.6% (52.0-57.1)	21.6% (19.0-23.2)	1.7% (1.1-2.4)	5.0% (3.9-6.2)
35-39	1,573	691/882	18.7% (16.9-20.8)	20.7% (18.7-22.7)	15.8% (14.0-17.7)	55.7% (53.2-58.2)	25.2% (23.1-27.5)	2.5% (1.8-3.4)	5.0% (3.9-6.2)
40-44	1,669	704/965	17.2 (15.4-19.1)	19.9% (18.0-21.9)	17.0% (15.2-18.8)	59.3% (56.9-61.6)	28.1% (26.0-30.3)	4.2% (3.3-5.3)	6.7% (5.5-8.0)
45-49	1,736	752/984	15.9% (14.2-17.7)	13.5% (12.0-15.2)	13.5% (11.9-15.2)	56.0% (53.6-58.3)	25.9% (23.9-28.1)	3.8% (3.0-4.8)	7.3% (6.1-8.6)
50-54	1,419	583/836	13.2% (11.5-15.0)	10.9% (9.3-12.6)	13.7% (11.9-15.6)	52.9% (50.3-55.5)	23.5% (21.3-25.8)	3.2% (2.3-4.2)	7.0% (5.8-8.5)
55-59	1,193	504/689	12.5% (10.7-14.5)	8.8% (7.3-10.6)	11.4% (9.7-13.3)	48.9% (46.0-51.7)	21.8% (19.5-24.2)	3.2% (2.3-4.3)	7.0% (5.7-8.6)
60-69	1,151	603/548	10.9% (9.1-12.8)	6.8% (5.4-8.4)	9.3% (7.7-11.1)	44.3% (40.0-47.9)	13.5% (11.5-15.6)	2.3% (1.6-3.4)	6.4% (5.1-8.0)
70+	975	379/596	7.5% (5.9-9.3)	1.4% (0.8-2.4)	5.1% (3.8-6.7)	38.1% (33.0-43.3)	4.2% (3.3-5.7)	1.0% (0.5-1.9)	4.1% (2.9-5.5)

Data are presented as a percentage (total number of “diagnosed” and “ probably having diseases”) and 95% confidence interval (95% CI).

### AD

The prevalence of a history of AD was 15.6% (those diagnosed, n = 2,375; those with a probable diagnosis, n = 535; Table 2). The prevalence of current symptoms peaked in those aged 5-9 years (16.6%), decreased with age, and was higher in males aged 45-49 years and ≥70 years (*P* < 0.05; Figure 1-b).

### FAs

The prevalence of a history of FAs was 12.3% (those diagnosed, n = 762; those with a probable diagnosis, n = 1,368) (Table 2). The prevalence of current symptoms according to age peaked in respondents aged 25-29 years (14.7%) and was higher in females aged 25-29, 40-44, 45-49, 50-54, and 60-69 years (*P* < 0.05; Figure 1-c).

### AR

The prevalence of a history of AR was 47.4% (Table 2). The prevalence of pAR was 27.5% (those diagnosed, n = 3,636; those with a probable diagnosis, n = 1,510). In total, 39.0% of the respondents reported a history of PO (those diagnosed, n = 5,059; probable diagnosis: n = 2,245). The prevalence of current symptoms of AR peaked in those aged 10-14 years (24.4%), decreased in those aged 20-29 years, increased in those aged ≥30 years, and was higher in males aged 5-9, 10-14, and 15-19 years, and in females aged 25-29 years (*P* < 0.05; Figure 1-d).

### AC

The prevalence of a history of AC was 19.5% (those diagnosed, n = 2,592; those with a probable diagnosis, n = 1,054) (Table 2). The prevalence of current symptoms according to age peaked in those aged 10-14 years (13.7%), decreased in those aged 20-29 years, increased again in respondents aged ≥30 years, and was higher in females aged 25-29, 45-49, 50-54, 55-59, and 60-69 years (*P* < 0.05; Figure 1-e).

### MAs and DAs

**Figure 2.**
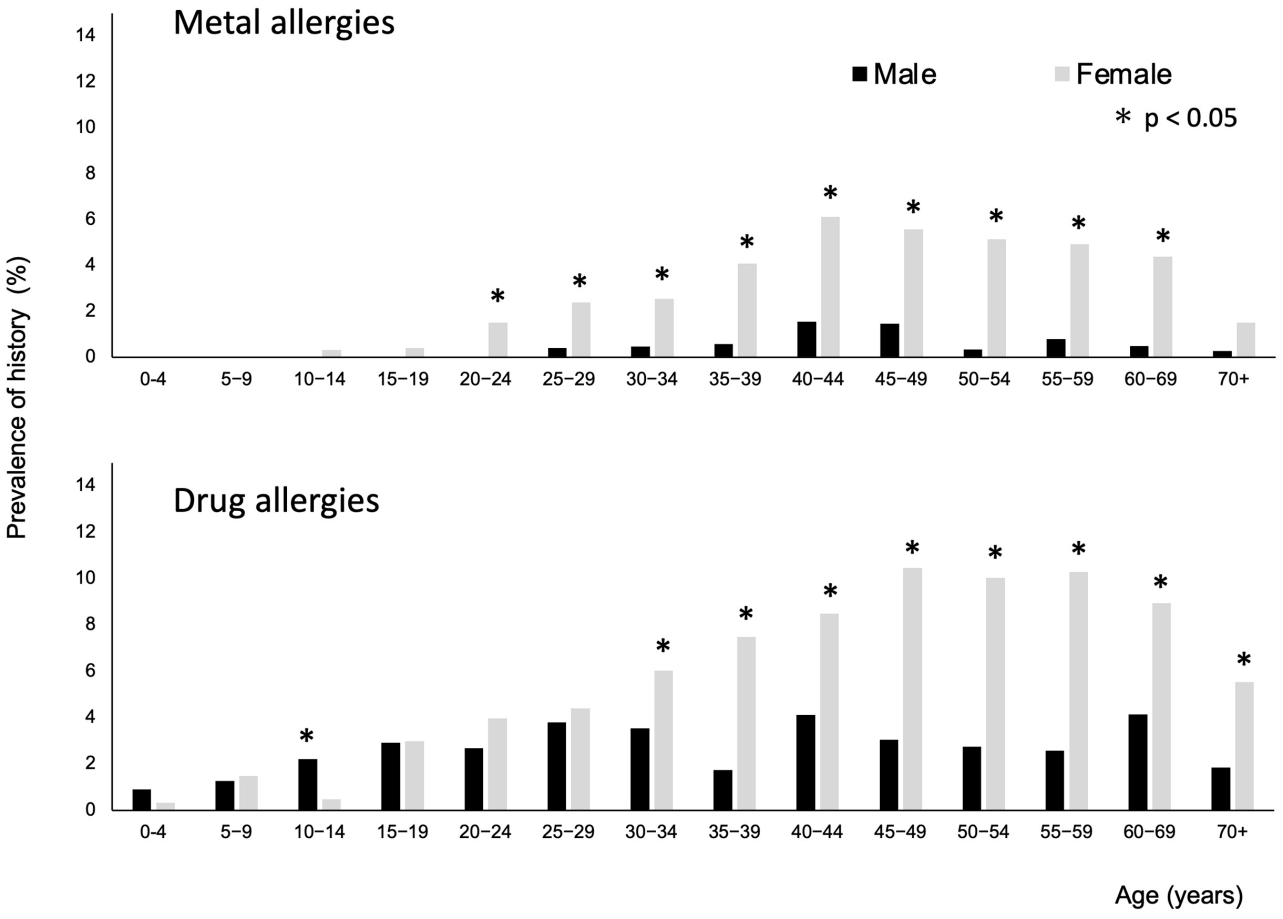
Prevalence of histories of metal and drug allergies. The percentage of respondents who reported a prevalence of histories of metal or drug allergies. *P*-values for sex differences in each age category were calculated using the chi-squared test.

Among all participants, 363 (1.9%) and 864 (4.6%) were reported to have been diagnosed with MAs and DAs, respectively (Table 2). The mean age of the patients with MAs was 45 years (min-max:10-91). The prevalence of a history of MAs was higher in females aged 20-69 years (*P* < 0.05; Figure 2). The mean age of the patients with DAs was 43 (range, 1-91) years. The prevalence of a history of DAs was higher in males aged 10-14 years and in females aged ≥30 years (*P* < 0.05).

### Overlap of allergic diseases

Among 11,626 respondents with allergic diseases, 4,326 had only one condition (37.2%), 3,312 had two (28.2%), 2,146 had three (18.4%), 1,073 had four (9.2%), 515 had five (4.4%), 221 had six (1.9%), and 33 had seven (0.3%) or more allergic diseases. Thus, 62.8% had two or more allergic diseases. Table 3 shows the overlap of allergic diseases. PO often coexisted with AC (79.9%) or pAR (69.8%). Regarding DAs, 8.9% coexisted with MAs. Conversely, for MAs, 21.2% coexisted with DAs.

**Table 3.** Overlap of Allergic Disease.

Coexisted allergic diseases (numerators)									
		BA	AD	FAs	pAR	PO	AC	MAs	DAs
Allergic diseases (denominator)	BA n = 2,747	-	33.2%	28.3%	50.0%	50.7%	31.9%	3.8%	9.0%
	AD n = 2,910	31.3%	-	34.0%	45.7%	52.3%	33.2%	3.4%	6.8%
	FAs n= 2,837	27.4%	34.8%	-	43.1%	57.9%	35.2%	3.6%	10.0%
	pAR n = 5,146	26.7%	25.8%	23.8%	-	69.8%	39.4%	3.3%	7.2%
	PO n = 7,304	19.0%	20.8%	22.5%	49.1%	-	39.8%	2.9%	6.7%
	AC n = 3 646	24.1%	26.5%	27.4%	55.7%	79.8%	-	3.4%	8.8%
	MAs n = 363	28.9%	27.5%	28.6%	47.6%	60.3%	34.9%	-	21.2%
	DAs n = 864	28.8%	22.9%	32.8%	43.4%	56.8%	37.3%	8.9%	-

Data are presented as percentages of the prevalence of coexisted allergic diseases (numerators) in each disease (denominator). BA: bronchial asthma, AD: atopic dermatitis, FAs: food allergies, pAR: perennial allergic rhinitis, PO: pollinosis AC: allergic conjunctivitis, MAs: metal allergies, DAs: drug allergies

### History of allergic disease by occupations

Table 4 shows the prevalence of histories of allergic diseases by occupation. There was no difference in the prevalence of a history of allergic diseases between physicians, nurses, and clerks, except for MAs. The prevalence of a history of MAs tended to be higher among female clerks (10.9%).

**Table 4.** History of Allergic Disease by Occupations.

**1) Male**									
**Occupation**	**n**	**Age mean** **(min-max)**	**Bronchial asthma**	**Atopic dermatitis**	**Food allergy**	**Allergic rhinitis**	**Allergic conjunctivitis**	**Metal allergy**	**Drug allergy**
Nurse	167	34.1 (21-60)	16.1% (10.9-22.6)	16.7% (11.4-23.3)	11.3% (7.0-17.2)	33.5% (26.4-41.2)	20.9% (15.1-27.9)	0%	5.9% (2.9-10.7)
Physician	932	43.2 (24-84)	22.5% (19.8-25.2)	22.1% (19.5-24.9)	12.1% (10.1-14.4)	35.4% (32.3-38.6)	35.4% (32.3-38.6)	1.0% (0.4-1.8)	5.3% (4.0-7.0)
Clerk	291	44.6 (21-69)	17.2% (1.3-2.2)	15.8% (11.8-20.5)	10.6% (7.4-14.8)	32.9% (27.6-38.7)	26.1% (21.2-31.6)	0.6% (0.1-2.5)	3.4% (1.7-6.2)
Medical technologist	102	43.3 (23-72)	18.6% (11.6-27.6)	13.7% (7.7-22.0)	10.7% (5.5-18.5)	33.3% (24.3-43.4)	21.5% (14.0-30.8)	0%	2.9% (0.6-8.4)
Pharmacist	99	39.3 (25-62)	21.1% (13.6-30.6)	24.2% (16.2-33.9)	13.1% (7.2-21.4)	32.3% (23.3-42.5)	28.2% (19.7-38.2)	1.0% (0-0.5)	8.0% (3.6-15.3)
Other	293	42.4 (21-72)	18.8% (14.5-23.7)	18.7% (14.2-23.4)	13.6% (9.9-18.1)	37.5% (32.0-43.4)	26.5% (21.6-32.1)	1.3% (0.4-3.5)	7.1% (4.5-10.7)
**2) Female**									
**Occupation**	**n**	**Age mean** **(min-max)**	**Bronchial Asthma**	**Atopic Dermatitis**	**Food allergy**	**Allergic rhinitis**	**Allergic conjunctivitis**	**Metal allergy**	**Drug allergy**
Nurse	2453	37.6 (20-67)	17.7% (16.2-19.3)	20.0% (18.4-21.6)	14.9% (13.5-16.4)	36.8% (34.9-38.8)	28.5% (26.8-30.4)	3.9% (3.2-4.8)	9.9% (8.8-11.2)
Physician	459	38.2 (24-70)	18.0% (14.5-21.7)	20.2% (16.7-24.2)	14.5% (11.3-17.9)	32.0% (27.8-36.5)	35.5% (31.1-40.1)	3.7% (2.2-5.9)	8.9% (6.5-11.9)
Clerk	1004	44.5 (21-67)	19.5% (17.1-22.1)	21.0% (18.5-23.7)	17.3% (14.9-19.7)	36.9% (33.9-39.9)	36.0% (33.0-39.0)	7.3% (5.7-9.1)	9.6% (7.8-11.6)
Medical technologist	204	40.2 (22-67)	19.1% (14.0-25.2)	18.6% (13.5-24.7)	15.6% (11.0-21.4)	31.3% (25.1-38.2)	34.3% (27.8-41.3)	4.9% (2.4-8.8)	6.3% (3.4-10.7)
Pharmacist	164	36.0 (24-64)	15.8% (10.6-22.4)	18.2% (12.7-25.1)	14.6% (9.6-21.0)	35.3% (28.1-43.2)	33.5% (26.4-41.3)	1.8% (0.4-5.3)	6.7% (3.4-11.7)
Other	805	43.0 (17-80)	18.7% (16.0-21.5)	21.3% (18.5-24.2)	17.7% (15.1-20.5)	37.7% (34.3-41.1)	37.8% (34.4-41.2)	4.9% (0.5-6.6)	10.9% (8.9-13.3)

Data are presented as a percentage (total number of “diagnosed” and “ probably having diseases”) and 95% confidence interval (95% CI).

### Allergic marches in children

**Figure 3.**
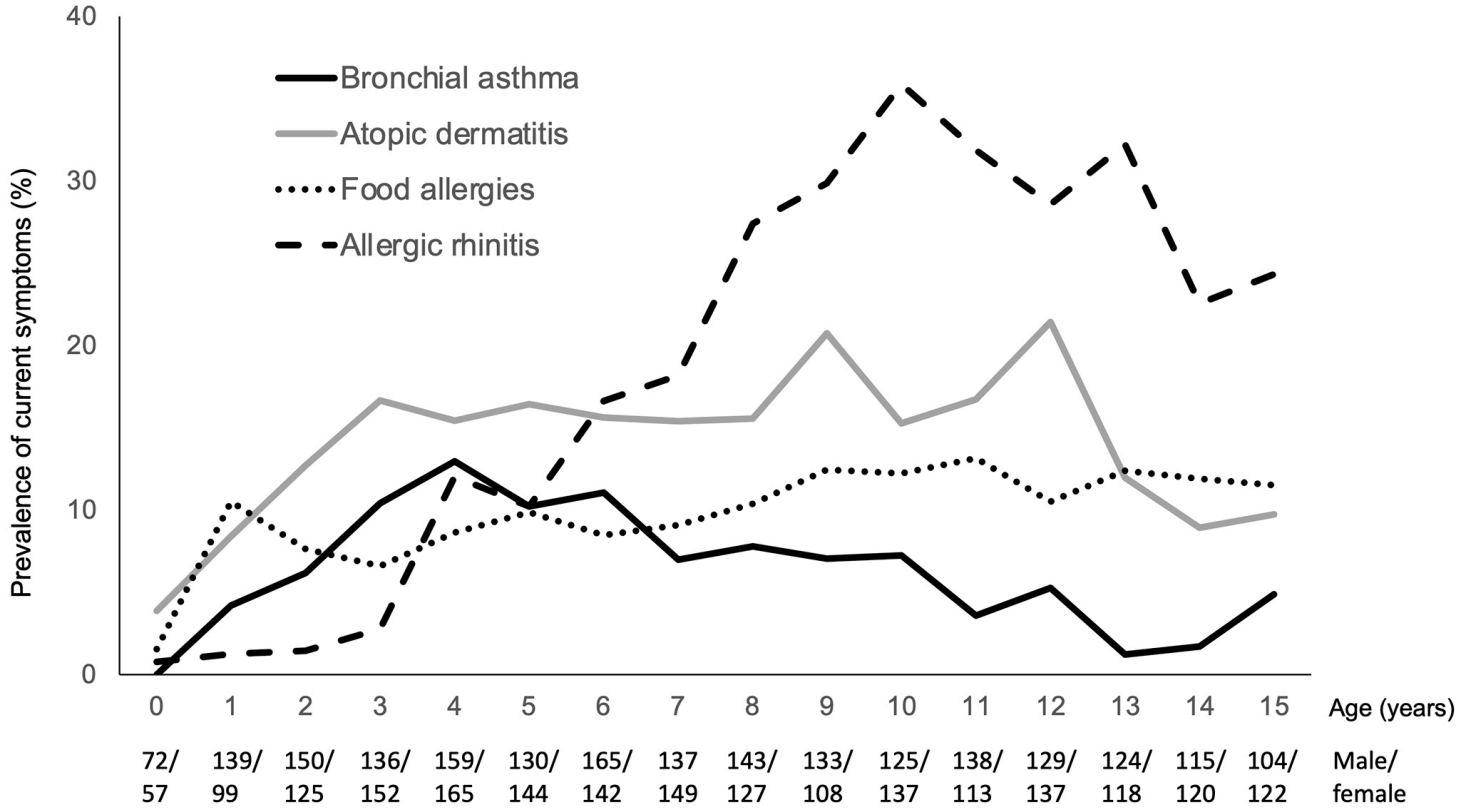
Allergic marches in children. Percentage of respondents who reported current symptoms of bronchial asthma, atopic dermatitis, food allergy, or allergic rhinitis in each age group from 0 to 15 years. The numbers of male and female respondents in each age group are shown below.

Figure 3 shows the prevalence of the current symptoms of BA, AD, FAs, and AR among children aged 0-15 years. AD is the most prevalent allergic disease at the age of 0 years, increasing to 15% at approximately 3 years of age. The prevalence of current symptoms of FAs was 10% at 1 year of age and remained at approximately 10% thereafter. The prevalence of current symptoms of BA increased until 4 years of age (12.7%) but decreased in older children. The prevalence of current symptoms of AR increased starting at 3 years of age. AR was the most prevalent allergic disease in children aged ≥6 years.

## Discussion

Using a designated allergic disease medical hospital network, we conducted a nationwide cross-sectional survey of allergic diseases across all ages to determine the prevalence of allergic diseases, including age-related changes and sex-related differences. These results suggest that approximately two-thirds of the Japanese population is likely to have allergic diseases.

The prevalence of current symptoms of each allergic disease varies with age. BA, AR, and AC peaked in childhood, declined among those aged 20-29 years, and then increased thereafter. AD peaked during childhood and gradually declined thereafter. The age distribution of MAs and DAs prevalence differed from that of other allergic diseases, peaking in adulthood.

When conducting a large scale survey, the method through which the presence of a disease is determined must be defined. Because allergic diseases are common and generally not severe, several patients do not visit a physician for diagnosis or treatment. Thus, this study included respondents who answered “Not diagnosed, but I (or my family members) probably have allergic diseases” to the question “Have you (or a family member) ever been diagnosed by a physician for an allergic disease?” as having an allergic disease, except for MAs and DAs. MAs and DAs were identified only when diagnosed by a physician to ensure accurate diagnosis when investigating their prevalence. Although 80% of respondents with BA or AD had been diagnosed by a physician, only 35.8% of children (aged 0-19 years) and only 28.7% of adults with FAs had been diagnosed by physicians (data not shown). This finding suggests that FA care is inadequate.

The prevalence of allergic diseases varies widely from country to country ^(4)^. Hence, the results of this survey should be compared with those of previous reports of allergy prevalence in Japan.

Most of the previous studies on the prevalence of BA in Japan have employed the ISAAC (International Study of Asthma and Allergies in Childhood) and ECRHS (European Community Respiratory Health Survey) questionnaires. The ISAAC questionnaire asks whether a respondent has experienced wheezing (whistling) ^(1)^. In 2015, a nationwide survey using the ISAAC questionnaire reported that the prevalence of wheezing was 10.2% in individuals aged 6-8 years (n = 42,176) and 8.2% in individuals who were 13-15 years (n = 36,243), which was similar to our data ^(5)^. The ECRHS Questionnaire is an international BA survey questionnaire used for individuals aged 20-44 years. In this questionnaire, a positive answer to “Have you had wheezing or whistling in your chest at any time in the last 12 months?” was employed to identify the presence of current symptoms ^(6)^. One survey that used ECRHS reported that the prevalence of current symptoms of BA in Japanese adults was 9%-10% in 2006-2007 ^(7)^.

Based on physicians’ examinations, the prevalence of AD in Japanese children was surveyed in 2000-2002 and was found to be 9.8% at 1.5 years of age, 13.2% at 3 years of age, 11.8% at 7 years of age, 10.6% at 12 years of age, and 8.2% in first-year university students ^(8)^. A similar study among adult university employees from 2006 to 2008 determined an AD prevalence of 10.2% in individuals aged 20-29 years, 8.3% in individuals aged 30-39 years, 4.1% in those aged 40-49 years, and 2.5% in those aged 50-69 years ^(9)^. The results of these studies are generally consistent with those of our study.

The prevalence of FAs varies widely between reports due to differences in disease definition and population samples. The prevalence of caregiver-reported FAs in a Japanese cohort study was 7.6%, 6.7%, and 4.9% among those aged 1, 2, and 3 years, respectively ^(10)^. Our study asked questions concerning the diagnosis and symptoms of FAs, including oral symptoms. The prevalence of FAs was found to be approximately 10% in those aged 1-19 years and 15% in those aged 20-50 years, which was higher than that in other reports, suggesting that the criteria used in this study to identify patients with FAs could have also identified patients with pollen-food allergy syndrome ^(11)^.

In 2019, a study surveyed the prevalence of AR among otolaryngologists and their family members in Japan ^(12)^. Diagnosis by an otolaryngologist is likely to involve a high degree of accuracy in terms of disease identification. The prevalence of pAR and PO across all ages was 24.5% and 42.5%, respectively (n = 19,859), which is similar to the results of our study.

Studies on the prevalence of MAs are rare. A survey of patients who visited a dermatologist revealed that contact dermatitis made up 3.92% of all skin diseases ^(13)^. There are few reports of prevalence studies on DAs, but systematic reviews of global data on DAs based on self-reports have been undertaken ^(14)^. An analysis of 53 publications, including 126,306 patients, showed that the prevalence of DAs was 8.3% (range 0.7%-38.5%) in 2016. DAs were the most prevalent in adult women, which is consistent with the results of our study. Our study is the first to survey the prevalence of MAs and DAs on a large scale in Japan.

Moreover, our study revealed variations in the prevalence of each surveyed allergic disease across all ages by sex. The prevalence of current symptoms of BA and AR was higher in male children, whereas that of FAs and AC was higher in adult females. Nevertheless, no sex-related differences in AD were observed during childhood. The prevalence of allergic diseases has previously been reported to be higher in males during childhood and to predominate among females thereafter ^(15), (16)^.

This survey showed that allergic diseases coexisted, and the overlapping rates varied depending on the disease. This may be related to differences in sensitized allergens, such as pollen and mites, in the development of each disease. The complication rate of DAs and MAs is also indicated in this study; however, it has rarely been reported in the previous literature. Hence, further analysis of the association of allergic diseases is required.

Although the survey methods and disease definitions differed between our study and previous studies, similar patterns in the prevalence of allergic diseases were observed.

Furthermore, our study revealed differences in the prevalence of allergic diseases during childhood. AD had the highest prevalence during infancy, followed by FAs and BA. These data seem to support a time course concerning allergic diseases in childhood, known as the allergic march ^(2)^. By contrast, AR is the most prevalent allergic disease in children aged >6 years. Yamamoto-Hanada et al. reported comparable results in their birth cohort study ^(9)^.

This national cross-sectional survey had several limitations. One limitation concerns selection bias because the respondents were employees of medical institutions who were likely to have been more familiar with allergic diseases than the general population. Allergic diseases are known to develop in association with occupation. Healthcare workers may be exposed to cleaning agents, disinfectants, latex, and various medications ^(17)^, but the results showed that there was no difference in the prevalence of a history of allergic diseases between physicians, nurses, and clerks, except for MAs (Table 4).

Second, participation in the survey was voluntary. Since the announcement methods used in each hospital vary, calculating an accurate response rate is challenging. Staff who had allergic symptoms or whose family members had allergic diseases might have responded to online questionnaires more often. Despite these limitations, our data showed a similar prevalence of each disease as previously reported. Recently, along with societal changes, conducting a nationwide survey has become difficult. The methods employed in our study may be useful for follow-up studies on the prevalence of allergic diseases.

In conclusion, approximately two-thirds of the Japanese population may have at least one allergic disease. Since the development of allergic diseases is related to genetic predisposition, environmental factors, culture, social background, and available treatment options, the prevalence of allergic diseases tends to change dynamically. Therefore, future epidemiological studies of allergic diseases should be conducted periodically.

## Article Information

### Conflicts of Interest

None

### Sources of Funding

This work was supported by a science research grant for research on allergic diseases and immunology from the Ministry of Health, Labour, and Welfare in Japan (grant number: 20FE2001).

### Acknowledgement

We would like to thank the staff of the allergic disease medical hospitals and their families for their participation in this study.

### Author Contributions

YI, TK, and YA wrote the manuscript. YI, TK, and KT collected the data, performed statistical analyses, and interpreted the results. All authors designed the study and read and approved the final manuscript.

### Approval by Institutional Review Board (IRB)

This study was approved by the ethics committee of Toyama University (ID: R2021089).
